# Effects of Adverse Events and 12-Week Group Step Aerobics on Sleep Quality in Chinese Adolescents

**DOI:** 10.3390/children10071253

**Published:** 2023-07-21

**Authors:** Yuwei Hu, Xiyan Duan, Zhuoran Zhang, Chunxia Lu, Yang Zhang

**Affiliations:** 1 College of Physical Education, Hunan Normal University, Changsha 410081, China; 0svald0hyw@hunnu.edu.cn (Y.H.); 202220152969@hunnu.edu.cn (X.D.); 2 Hunan Sports Vocational College, Changsha 410081, China; 3 Independent Person, Windermere, FL 34786, USA; yzhang68@crimson.ua.edu

**Keywords:** anxiety, exercise, GAMLSS, Pittsburgh Sleep Quality Index, sleep disorders

## Abstract

Background: In China, sleep disorders have become a public health concern. This study aimed to model the relationship between adverse events and sleep quality, as well as the effect of group step aerobics on sleep quality. Methods: The modeling was built on surveying 2760 16–19-year-old adolescents. The Pittsburgh Sleep Quality Index (PSQI) was used to evaluate sleep quality, and the Adolescent Self-rating Life Events Checklist (ASLEC) was used to evaluate adverse events. Adolescents with sleep disorders (PSQI ≥ 8) were randomized into the control (*n* = 26) and exercise (*n* = 26) groups. The exercise group participated in 12-week step aerobics, and the 300 min weekly volume is compliant with the WHO physical activity guidelines. Results: The double Poisson distribution was chosen to fit the data. ASLEC had a nonlinear relationship with the PSQI. Participants in the exercise group slept better (*p* < 0.05) from the eighth week until the end of the study. A random adolescent, therefore, has a 92.5% probability of experiencing improved sleep quality after 12 weeks of step aerobics. Conclusions: Intervention should be implemented before adverse events accumulate. An active lifestyle should be a preparedness strategy for increasing the resilience of adolescent mental health in the face of adversity.

## 1. Introduction

More than 300 million people in China have sleep disorders, especially among the middle class and those performing intellectually demanding work [1]. Nearly 26% of Chinese adolescents experience sleep disorders [2], which is much higher than the global average [3]. Given the importance of sleep to health and the progression of various noncommunicable diseases [4], the high prevalence of sleep disorders poses a threat to public health in contemporary Chinese society.

Sleep disorders in adolescents are often caused by non-pathological factors. For example, excessive smartphone use at night has been a significant contributor to sleep disturbances [5]. Frequently, event-induced social anxiety is an important driver for this cohort’s sleep behavior change. From the perspective of evolutionary biology, university-aged young adults experience the greatest stress, including selective reproduction stress. As a young adult who just begins an independent life with various daily stressors, negative emotions induced by adverse events can cause significant psychosocial and behavioral disturbances and develop into sleep disorders [6]. Consequently, adverse events are a primary cause of stress, anxiety, depression, and sleep problems among this cohort.

Adverse events are unquestionably associated with an increased risk for and persistence of sleep disorders. However, current research only addresses their correlation, whereas their interactive effects remain to be clarified. Adverse events may accumulate throughout a person’s life stages, and little is known about their combined effects on sleep quality. Similarly, adverse events are immediate stimuli for negative emotions, and they may also recur [7], resulting in a lasting impact on sleep quality. Exploring their relationships can lead to more effective interventions not only for sleep health but also for psychological disorders such as depression. In this regard, regression analyses can deepen our comprehension of their interplay beyond simple correlational analysis. It is now possible to model such complex relationships, owing to advanced statistical techniques, such as the Generalized Additive Models for Location, Scale, and Shape (GAMLSS) [8]. Therefore, the first objective of this study was to explore the relationship between adverse events and sleep quality using a distributional regression approach.

This study further examined exercise on sleep quality. It is widely accepted that exercise ameliorates pathophysiological and psychiatric sleep disorders [9]. In China, step aerobics has gained popularity since the COVID-19 control period because of its home-based nature for convenient participation, low equipment requirements, and trending mobile-based live fitness coaching. Previous research has shown that 10 weeks of step aerobics can enhance sleep quality and melatonin levels in postmenopausal women with sleep difficulties [10]. This benefit can be attributed in part to its capacity to reduce trait anxiety [11] and social anxiety [12] in young adults. To our knowledge, its efficacy in alleviating sleep disorders in young cohorts has not been examined. In light of this context, adolescents with sleep disorders were invited to participate in step aerobics to quantify its effect on their sleep quality. Using a WHO-recommended exercise volume [13], we examined the duration required to improve sleep quality.

Overall, the purpose of this study was to expand theoretical and practical foundations for addressing the increasing prevalence of sleep disorders among Chinese adolescents. It was hypothesized that adverse events are an important cause of sleep disorders and that 12 weeks of step exercise is required to improve sleep health.

## 2. Materials and Methods

### 2.1. Participants

The study was conducted in accordance with the Declaration of Helsinki and approved by the Ethics Committee of the Hunan Normal University. Informed consent was obtained from all participants or legal guardian(s) of minor participants. Table 1 provides details about their demographics.

In the modeling study, a cluster randomized sampling was utilized. An online survey was sent out to 3500 university students from five Changsha-based universities. There were 3490 valid surveys returned. After filtering for the desired age range, 2760 surveys were used for modeling.

In the exercise study, the minimum sample size needed for a two-group randomized controlled trial was estimated a *priori*. Banno and colleagues meta-analyzed the effect of exercise on sleep health in a healthy population [14]. We extracted their data and calculated the effect size for sleep quality. Using G*Power version 3.1, it was determined that 48 participants were required for a two-group experiment with an effect size of 0.971 and a power of 0.95 (one-tailed). In the healthy Chinese population, adolescents with a Pittsburgh Sleep Quality Index (PSQI) [15] scores of seven or higher are regarded as presenting sleep disorders [16]. To avoid marginal effects in treating sleep disorders, this study enrolled adolescents with a PSQI score of eight or above. The other inclusion criteria were as follows: no psychological counseling within the past three months; no history of psychosis; no hallucinations, thought disorders, delusions, or other psychotic symptoms; no history of physical illnesses that prevent vigorous exercise. We recruited 64 eligible volunteers, each of whom completed assessments and exercise training over the course of the 12-week study. After filtering for the desired age range, data from 52 participants (26 in each group) were used for the present analysis.

### 2.2. Online Survey

The survey inquired about demographic information, including sex, age, BMI, ethnicity, and hukou (Chinese household registration). In addition to the basic demographics listed in Table 1, this population consisted of 81.6% Han Chinese, 18.4% minority Chinese, 55.1% rural residents, and 44.9% urban residents. The main components of the online survey are described as follows:

The Adolescent Self-rating Life Events Checklist (ASLEC) [17] was used to assess adverse events that occurred within the past 12 months. The ASLEC includes 27 items, and individual item scores are added to obtain the global score, with a higher score indicating a greater number of stressful life events experienced over the past year. In this study, Cronbach’s alpha was 0.942, and the KMO value was 0.961.

The PSQI measures seven components of sleep health, and each component is assessed on a 3-point scale, with a higher score indicating poorer sleep quality. When all component scores are added together, the global score indicates the individual’s overall sleep health for the previous month. In this study, Cronbach’s alpha and KMO values were 0.812 and 0.799, respectively.

### 2.3. Exercise Protocol

Participants were randomly assigned to the control group and the exercise group. Participants in the control group were instructed to engage in only free-living activities for the duration of the study, while those in the exercise group engaged in group-based step aerobics. The exercise group performed step aerobics on a 15-cm tall platform for 75 min per session, four times per week. All exercise was performed indoors. A university professor of dance choreographed step aerobics, which incorporated arm swings, jumps, turns, and varying footwork. To accomplish the desired exercise benefits and motivation, a custom playlist of Chinese pop music with a medium tempo (110–120 beats per minute) was compiled and utilized during training. Led by a dancing coach, the initial 10 min of a training session consisted of slow-paced stepping and upper-body exercises to elevate the heart rate. This was followed by 60 min of exercise and 5 min of stretching and cool-down. The average heart rate varied between 120 and 160 beats per minute.

Participants were instructed to report the ASLEC, PSQI, and the Self-rating Anxiety Scale (SAS) [18] at the baseline, 4th week, 8th week, and conclusion (T + 24 h) of the 12-week exercise. The SAS is an instrument for assessing anxiety levels. The scale consists of 20 items, and item scores are added to produce the global score, with high scores indicating a higher anxiety level. In this study, Cronbach’s alpha and KMO values were 0.74 and 0.90, respectively.

### 2.4. Modeling Approach

Regression analysis was performed using the GAMLSS package version 5.4–12 in the RStudio version 2023.03.1 Build 446. Based on the data structure of the response variable, which consists of discrete values and is short of zero (0.80% of 2760 observations), we compared 30 explicit discrete distributions available in the GAMLSS and selected the distribution with the smallest value of the Schwarz Bayesian criterion [19] as the marginal distribution for subsequent modeling. A stepwise strategy, as described by Ramires and colleagues [20], was used for additive term selection. The penalty used in the fitting algorithm was based on the Schwarz Bayesian criterion (i.e., *k* = 8.16), and the quantitative covariables were smoothed using a local maximum likelihood penalized B-splines method. The randomized quantile residuals were used to examine the goodness-of-fit of the conditional distribution.

The generalized likelihood ratio test statistic was used to compare the one-parameter Poisson model (using the additive smoothing terms in the conditional distribution) with the GAMLSS fitted model. This comparison based on the difference in the global deviance between Poisson and fitted models is equivalent to testing the null hypothesis that the response variable’s dispersion (*σ*) is not zero [21].

### 2.5. General Statistics

GraphPad Prism version 9.0.0 was used to analyze the data from the exercise study. Given that none of the outcome measures follow the Gaussian distribution, non-parametric statistics were used for analysis as well as interpretation. The main effect of exercise training was compared using the Mann–Whitney test, while the main effect of time was compared using the Kruskal–Wallis test. When significant differences were identified, Dunn’s multiple comparisons test was applied. For broad dissemination of the results to the general public without a scientific background, we interpreted the effect size of exercise training using the non-parametric, probability-based *A* statistic [22]. The alpha level was set at 5%.

## 3. Results

### 3.1. Effect of Adverse Events on Sleep Quality

The *p*-value for the generalized likelihood ratio test statistic was less than 0.001, thereby justifying the beyond mean (location) statistical modeling. After fitting the data to 30 explicit GAMLSS distributions, the two-parameter double Poisson (DPO) distribution [23] was chosen as the marginal distribution of the PSQI score. DPO has an approximate mean of $\hat{\mu }$ and an approximate variance of $\hat{\mu }$ × $\hat{\sigma }$. The resulting model from the stepwise strategy is given as follows:

(1) $$Y\;\sim \;\mathrm{DPO}\;(\hat{\mu },\;\hat{\sigma })\;\hat{\mu }\;\text{= exp [1.3655 + 0.0142 × pb (ASLEC; edf, 4.983; λ, 4327.176)]}\;\hat{\sigma }\;\text{= exp [0.2579 − 0.0071 × pb (ASLEC; edf, 8.429; λ, 28.729)],}$$

where edf is the effective degree of freedom and λ is the smoothing hyper-parameter.

**Figure 1:**
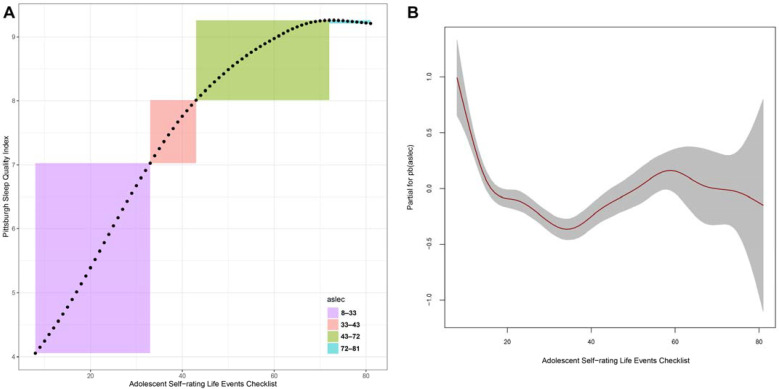
A double Poisson model for the Pittsburgh Sleep Quality Index (PSQI) score. (**A**) location plot of adverse events on PSQI; (**B**) partial effect of adverse events on PSQI.

Using the equation, Figure 1A depicts the resultant location plot based on the ASLEC data range in this sample. The effect of ASLEC on PSQI was nonlinear: the PSQI score increases in tandem with ASLEC scores between 8 and 33, where an ASLEC score of 33 approximately corresponds to the PSQI threshold score of 7; then, their relationship remained approximately linear between ASLEC scores of 33 and 43; after an ASLEC score of 43, the PSQI score progressively plateaus. Of note, between ASLEC scores of 74 and 81, the PSQI score decreased from 9.26 to 9.21, as estimated by the model. This behavior may be the result of few data within this range (*n* = 18), thereby diminishing the statistical precision. Figure 1B shows that ASLEC influenced the PSQI score dispersion. ASLEC had a multi-level partial effect on the scale parameter: between ASLEC scores of 8 and 34, sigma decreased nonlinearly, followed by an approximately linear increase up to an ASLEC score of 59 and then an approximately linear decrease. The widened band (Figure 1B’s shaded area) for ASLEC scores of 59 and higher was caused by a smaller sample size; consequently, the accuracy in this range should be viewed with caution.

**Figure 2:**
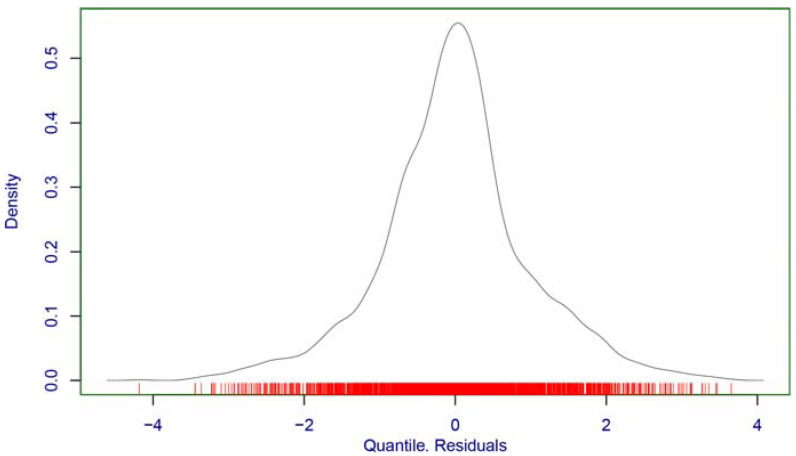
Density estimate of the DPO model’s randomized quantile residuals.

Concerning residual analyses (Figure 2), the model had a mean of −0.00022, variance of 0.9983, moment-based skewness of 0.0247, kurtosis of 4.0926, and Fiberfill correlation coefficient of 0.9915. This indicates inadequacies in the model’s shape parameters. This is, however, understandable given that DPO has only two parameters, making it impossible to model the shape of the data. Given that this limitation cannot be overcome with existing explicit GAMLSS distributions, the DPO model is adopted.

### 3.2. Effect of Step Aerobics on Sleep Quality

Neither dropout nor injuries as a result of step aerobics participation occurred during the 12 weeks. Table 2 presents the main effects of exercise and time. For the control group, there was no change in PSQI scores over the course of the study. The PSQI score of the exercise group became significantly lower beginning in the eighth week, and this trend persisted through the conclusion of the study. In other words, a random healthy person participating in 8-week step aerobics had a 68.5% chance of experiencing improved sleep quality than the nonactive group, and this probability jumped to 86.4% should exercise training last for 12 weeks. The main time effect indicates that there was a 92.5% probability that a random person would experience improved sleep quality after 12 weeks of step aerobics.

Meanwhile, there was no change in the ASLEC score in either group. After 12 weeks of step aerobics, the main training effect indicates a 76.8% probability that a random person would experience less anxiety than the nonactive group. Overall, there was an 80.4% probability that a random person would experience less anxiety.

## 4. Discussion

Adverse events are a known cause of poor sleep quality [24]. Campbell and colleagues reported that poor sleep quality preceded exams in university students [25], reflecting that a single adverse event can affect sleep quality. Nonetheless, a person often encounters multiple adverse events in every phase of life [26]. Here, we show that the rate and magnitude of a pattern change in sleep are not constant. Using a distributional regression, we observe a nonlinear relationship between adverse events and sleep quality in a university student population. Above a certain threshold, such as ASLEC scores above 43 in healthy adolescents, the deterioration in sleep quality appears to eventually reach a plateau despite a further increase in adverse events. From a psychiatric perspective, adverse events have an impact on mental well-being [27], triggering short-term anxiety, long-term self-esteem issues, and more severe recurring depression symptoms. Many adverse events for young adults, such as academic stress and peer pressure, are not disease-related; as a result, changes in mental well-being heighten negative emotions and consequently affect their sleep quality. Previous research found that mental stress levels increase steadily during adolescence before “stabilizing” [28], albeit at an unstable level, during the transition to adulthood. This may be the result of coping mechanisms as young adults learn to manage stress. Consequently, this pattern shift in the mental development trajectory may account for the plateau in poor sleep quality. Modern studies bring a new perspective on the role of chronic stress resulting from adverse events in the development of physiological abnormalities. It is hypothesized that chronic stress causes the down-regulation of glucocorticoid receptors [29], which leads to various pathological developments, such as mood disorders. It is also plausible that cumulative stress triggers abnormal physiology and mental cross-pathways [30] and that such glucocorticoid receptor resistance reaches a state of physiological stability, albeit abnormal, thereby limiting further deterioration in sleep quality. Therefore, our finding not only provides new insights regarding the sleep quality curve but also points out a direction for future research to determine underlying mechanisms.

It is useful to distinguish the two-way relationships between adverse events and sleep quality. This study shows adverse events have a nonlinear effect on sleep quality: as adverse events accumulate, sleep quality declines. However, the other way around is not guaranteed: when adverse events subside, sleep quality may not return to normal. There are many types of adverse events. Acute stress disorder and, consequently, poor sleep quality can be caused by an acute health condition or injury, and once these events pass, sleep health will eventually return to normal. However, more severe adverse events may have lasting repercussions and cause psychological trauma, and as time goes on, people, particularly children, who encounter such events may develop chronic sleep disorders. As a result, adverse events can become subconscious in one’s thinking and may never fade. For example, children who grow up in unfavorable family environments (e.g., substance abuse or divorce) may suffer from sleep disorders into adulthood [31]. Children who have been sexually abused are often forced to endure a lifetime of victimization, and even 10 years after the occurrence of damaging events, severe sleep disorders persist [32]. Our findings should strongly alert family members, schoolteachers, and clinicians to take decisive action when suspicious behavior patterns (e.g., insomnia, daytime sleepiness) emerge among children and adolescents to help mitigate adverse events from becoming long-lasting traumas.

Previous research on the effects of demographic factors on sleep quality relied on a traditional regression method, and cross-factor comparisons were typically based on correlational analysis and individual factor-level comparisons. In this study, all covariables were taken into account concurrently to determine the regression relationship, which provides a more thorough method for determining their collective roles in sleep quality. Of note, our null results regarding sex, social demographics, and BMI do not deviate from earlier studies [33,34]. The point is that when all factors are considered, it is possible to conclude that adverse events trigger sleep disorders, highlighting the methodological sensitivity of using a modern distributional regression. Meanwhile, it may be inaccurate to underplay a complex issue, such as the sequential link between low socioeconomic status (including gender inequality in some cultures), development of obesity, chronic low-grade inflammation, decreased melatonin levels, and progressive deterioration of sleep quality. Future research is needed to apply the current method to a diverse population.

In this randomly sampled population, 44.9% of the university students exhibited inadequate sleep quality. This result is in line with other population studies [35]. The high prevalence of sleep disorders in this cohort is compelling evidence that sleep disorders are now a public health concern in China. Based on the location model, a small accumulation of minor unpleasant events can influence sleep quality. From an ethical standpoint, we chose exercise as a proactive intervention, which has a preventative effect on depressive symptoms [36] and a therapeutic effect on sleep disorders [9]. Given the evidence supporting a higher volume for better sleep quality [37], we adopted the upper band of exercise volume (300 min per week) recommended by the WHO for this age group. The outcomes are both reassuring and concerning. On the one hand, sleep quality improved in the eighth week, and this beneficial effect was even more obvious at the 12-week mark. According to the anxiety level, exercise was effective at reducing anxiety, which played a role in sleep health in this sample. These findings are consistent with previous research on the effects of exercise on anxiety and sleep quality [38]. This study shows that step aerobics can be prescribed to mitigate sleep disorders.

On the other hand, even though the exercise group showed a significant improvement in sleep quality, the overall score remained above the cutoff for sleep disorders, indicating that the stresses from adverse events were not temporary and that even 300 min of moderate-intensity exercise per week for 12 weeks may not be enough to restore normal sleep. Thus, we can conclude that the duration of exercise for this group should exceed 12 weeks and that additional psychological counseling is needed as an intervention, particularly for young adults experiencing severe adverse events. Moreover, even though exercise participation was voluntary and participants had the freedom to withdraw without any form of discrimination in this study, all participants were verbally encouraged to continue the exercise intervention. Therefore, this intervention may be considered supervised training. This becomes problematic in real-world circumstances. It is known that exercise adherence is a challenge for promoting and sustaining an active lifestyle. In addition, the more depressed a person is, the less likely they are to engage in structured physical activity [39], thereby creating a vicious cycle of adverse events, depression, decreased physical activity, sleep disorders, and more depression. Consequently, the actual effect of exercise may differ from the current experimental outcomes.

Finally, there are a few noteworthy limitations. First, we sampled university students because they are in a period of transition from adolescence to adulthood and are exposed to daily stresses and adverse events. While this sample provided reasonable statistical power to model the nonlinear relationship, it has a limited age span that only includes a portion of the adolescent age range. In light of this, our distributional regression model should not be regarded as the final model, and the framework presented in this work can serve as a point of reference for future studies encompassing a broader age range. In our view, differentiated maturational development of coping skills can influence the magnitude of adverse events on sleep quality. Typically, adolescents at a young age have poor stress management and, as a result, exhibit more convergent (i.e., less variable) responses to adverse events. As young people mature, they progressively develop adaptive coping strategies for negative emotions [40], whereas this development is differentiated by many factors [41], resulting in divergent responses to adverse events. Accordingly, we hypothesize that age may be an additional factor influencing sleep quality. Likewise, this population can also be regarded as a mono-social background group (e.g., university students living in residence halls). These limitations necessitate the inclusion of a more diverse population in future research to verify and/or extend our findings. Second, the poor tail fit from the DPO model suggests a lack of control over the shape parameters and/or the existence of additional influencing factors that were not accounted for when determining the location, scale, and shape of the PSQI distribution. This methodological inadequacy warrants more research. Given that none of the terms selected for the distributional parameters are unique to the Chinese population, they may have universal applicability, and we encourage researchers from other nations to examine additional covariables (e.g., anxiety, depression, stress) to clarify this issue.

## 5. Conclusions

Adverse events have a nonlinear driven effect on the sleep quality of Chinese adolescents in our sample. The nonlinear relationship suggests that intervention strategies should be implemented as soon as a single adverse event occurs, given that such effects on sleep quality are cumulative and that certain adverse events have long-lasting psychological consequences through adulthood. This study further shows that a 12-week exercise program at the WHO-recommended volume has a 92.5% probability of enhancing sleep quality in healthy adolescents; thus, routine exercise should be widely campaigned as a lifestyle modification for school-aged adolescents. Overall, this study expands our understanding of the socio-emotional factor that results in adolescent sleep disorders, and our empirical findings endorse promoting active living as a useful method for alleviating insomnia among Chinese adolescents.

## Figures and Tables

**Table 1:** Summary of participants’ demographics.

Category	Modeling Study (*N* = 2760)	Exercise Study (*N* = 52)
*n*, male	1170	13
*n*, female	1590	39
*M*, age (yrs)	18.4	18.4
*n*, 16 yrs	4	0
*n*, 17 yrs	200	4
*n*, 18 yrs	1222	25
*n*, 19 yrs	1334	23
*M*, BMI (kg/m^2^)	20.6	20.9

Note. BMI—body mass index.

**Table 2:** Results of 12-week exercise intervention.

Time	PSQI				SAS				ASLEC			
	E	C	*p* ^1^	*A* (%)	E	C	*p* ^1^	*A* (%)	E	C	*p* ^1^	*A* (%)
T0	10.0	9.0	0.09	36.4	55.0	51.0	0.05	34.1	43.5	43.5	0.70	46.8
T4	10.0	9.0	0.18	39.4	53.5	50.0	0.10	36.8	40.0	42.5	0.91	51.0
T8	9.0 **	9.5	0.02	68.5	51.0	51.0	0.56	54.8	39.0	43.5	0.37	57.3
T12	8.0 **^†^	9.5	<0.001	86.4	41.5 **	52.5	<0.001	76.8	40.0	41.5	0.37	57.3

Note. Data are expressed as the median. *A*—*A* statistic; ASLEC—Adolescent Self-rating Life Events Checklist; C—control group (*n* = 26); E—exercise group (*n* = 26); PSQI—Pittsburgh Sleep Quality Index; SAS—Self-rating Anxiety Scale. *p* ^1^—between-group *p*-value; ** within-group *p*-value < 0.01, vs. T0; ^†^ within-group *p*-value < 0.05 vs. T8.

## Data Availability

The data are accessible with approval by the Hunan Normal University. Individual investigators may reach out directly to the Hunan Normal University to apply for those data access approvals. The authors are unable to share these data with individuals outside of the designated research team members.
